# Why Do Bi-Factor Models Outperform Higher-Order *g* Factor Models? A Network Perspective

**DOI:** 10.3390/jintelligence12020018

**Published:** 2024-02-04

**Authors:** Kees-Jan Kan, Anastasios Psychogyiopoulos, Lennert J. Groot, Hannelies de Jonge, Debby ten Hove

**Affiliations:** 1 Research Institute of Child Development and Education, University of Amsterdam, 1018 WS Amsterdam, The Netherlands; a.psychogyiopoulos@uva.nl (A.P.);; 2 Department of Educational and Family Studies, Vrije Universiteit Amsterdam, Van der Boechorstraat 7, 1081 BT Amsterdam, The Netherlands

**Keywords:** bi-factor modeling, higher-order *g* factor modeling, psychometric network modeling

## Abstract

Bi-factor models of intelligence tend to outperform higher-order *g* factor models statistically. The literature provides the following rivalling explanations: (i) the bi-factor model represents or closely approximates the true underlying data-generating mechanism; (ii) fit indices are biased against the higher-order *g* factor model in favor of the bi-factor model; (iii) a network structure underlies the data. We used a Monte Carlo simulation to investigate the validity and plausibility of each of these explanations, while controlling for their rivals. To this end, we generated 1000 sample data sets according to three competing models—a bi-factor model, a (nested) higher-order factor model, and a (non-nested) network model—with 3000 data sets in total. Parameter values were based on the confirmatory analyses of the Wechsler Scale of Intelligence IV. On each simulated data set, we (1) refitted the three models, (2) obtained the fit statistics, and (3) performed a model selection procedure. We found no evidence that the fit measures themselves are biased, but conclude that biased inferences can arise when approximate or incremental fit indices are used as if they were relative fit measures. The validity of the network explanation was established while the outcomes of our network simulations were consistent with previously reported empirical findings, indicating that the network explanation is also a plausible one. The empirical findings are inconsistent with the (also validated) hypothesis that a bi-factor model is the true model. In future model selection procedures, we recommend that researchers consider network models of intelligence, especially when a higher-order *g* factor model is rejected in favor of a bi-factor model.

## 1. Introduction

To date, four out of the ten most-cited papers published in the *Journal of Intelligence* focus on bi-factor modeling to determine the structure of intelligence (Beaujean 2015; Cucina and Byle 2017; Eid et al. 2018; Morgan et al. 2015). What insights can we glean from these publications when we try to integrate the findings? First, in more than 90 percent of direct comparisons between bi-factor models and higher-order *g*-factor models, the bi-factor model outperforms its higher-order competitor statistically (Cucina and Byle 2017). Second, bi-factor modeling has raised concerns (Eid et al. 2018; Morgan et al. 2015) (see, also Bonifay et al. 2017; Bornovalova et al. 2020; Decker 2020; Greene et al. 2019; Hood 2008; Murray and Johnson 2013; Van Bork et al. 2017; Zhang et al. 2020). As Morgan et al. (2015) notes, for example, fit measures may show ‘statistical bias’ against higher-order *g* factor models in favor of bi-factor models. Such (potential) bias is one of the reasons why models of intelligence should be evaluated not only through statistical comparison, but also on conceptual and theoretical grounds (Morgan et al. 2015) (see also, Murray and Johnson 2013; Schmank et al. 2019). Finally, such grounds may have been a reason for some researchers approaching bi-factor solutions with caution (Morgan et al. 2015), but, for others, these grounds may have been a reason to continue to promote them (Beaujean 2015; Cucina and Byle 2017).

We conclude that despite the well-established better fit of bi-factor models of intelligence, the field seems to be divided over the appropriateness and usefulness of bi-factor modeling. Drawing inspiration from the fifth and sixth top-10-cited papers in this journal (Van der Maas et al. 2017, 2014), we believe that adding a network perspective on intelligence can contribute to the discussion, especially when combined with a series of illustrative data simulations.

Data simulations were also the basis of the aforementioned caution concerning the better fit of bi-factor models as compared to higher-order factor models. Morgan et al. (2015) employed the following setup and logic. First, they generated data according to both bi-factor models and higher-order *g* factor models. Next, they fitted both types of models to all generated data sets and compared the obtained fit statistics. From this comparison, the authors concluded that bi-factor models tend to be the best-fitting model, irrespective of whether the true model was the bi-factor model or the higher-order *g* factor model. Thus, they reasoned that if the generating mechanism in the population were a higher-order *g* model in reality, it would be difficult to show, on the basis of fit statistics alone, that this model should be preferred over the bi-factor model, as the latter tends to fit better.

The study of Morgan et al. (2015) seems to be inspired by an earlier simulation study carried out by Murray and Johnson (2013). These authors employed a slightly different setup, but reached similar conclusions. Instead of fitting the exact true (higher-order *g* factor) model to the simulated data, they fitted a model that was *close* to the true model. Next, they compared the fit statistics of this slightly different (higher-order *g* factor) model with those of a bi-factor model in which the fitted model was nested. Thus, in this study, the competition was actually between two untrue models. For example, while the true model was a higher-order *g* factor model that included a limited number of cross-loadings, the fitted higher-order *g* factor model lacked these cross-loadings, making it locally and only slightly misspecified. Ultimately, this misspecification led to the rejection of the fitted higher-order *g* factor model, favoring the bi-factor model. Conceptually, however, the rejected higher-order *g* factor model was closer to the true model than the bi-factor model was.

From these simulation studies, we conclude that although the simplest explanation for why bi-factor models outperform their higher-order competitors might be that these models represent the true data-generating mechanism, or a model close to it, alternative explanations for the outperformance exist and should be seriously considered by the intelligence research community. Statistical bias is one such explanation and an important one, but it may not be the only one. Recent network studies into the structure of intelligence provide another potential explanation:

[In a network model, it is] in principle possible to decompose the variance in any of the network’s variables into the following variance components: (1) a general component, (2) a unique component, and (3) components that are neither general nor unique (denoting variance that is shared with some but not all variables). A bi-factor model can then provide a satisfactory statistical summary of these data.

(Kan et al. 2020, p. 4)

Here, we add that, in such a case, a bi-factor model may outperform a higher-order *g* factor model because the latter is nested within the bi-factor model (Yung et al. 1999), and therefore can only fit worse than the bi-factor model (though perhaps not significantly so). Essentially, this network explanation aligns with Murray and Johnson (2013)’s argument that when fitted models differ from the true model, and these fitted models concern nested models, the most complex of these models will have a higher likelihood of fitting the data. In more technical terms, the more complex model has a higher so-called “fit propensity” (Falk and Muthukrishna 2021).

The aim of the present study is to investigate the validity and plausibility of the network explanation as to why bi-factor models outperform higher-order *g* factor models, while controlling for the rivalling explanations that (1) the bi-factor model is the true model, and (2) the fit indices are biased against the higher-order *g* factor model in favor of the bi-factor model. Because the majority of comparisons between higher-order *g* factor models and bi-factor models were conducted using the Wechsler scale batteries, specifically for adult (Cucina and Byle 2017), we used the Wechsler Adult Intelligence Scale (WAIS–IV; Wechsler 2008) as our starting point. That is, we first provide a brief overview of the relevant psychometric properties of this battery. These properties encompass both factor analytic and network properties. Next, based on these properties, we present a Monte Carlo simulation study to assess the validity and the plausibility of each of the three rivalling explanations. Finally, we discuss the implications of our results for future simulation studies, as well as empirical research on the structure of intelligence.

## 2. The WAIS–IV; Factor-Analytical versus Psychometric Network Perspectives

The WAIS–IV consists of 15 subtests: Similarities (SI), Vocabulary (VO), Information (IN), Comprehension (CO), Block Design (BD), Matrix Reasoning (MA), Visual Puzzles (VP), Picture Completion (PC), Figure Weights (FW), Digit Span (DS), Arithmetic (AR), Letter–Number Sequencing (LN), Symbol Search (SS), Coding (CD), and Cancellation (CA). Table 1 provides a brief description of each of these subtests. According to the WAIS–IV manual, these subtests target four cognitive constructs: Verbal Ability (V), Perceptual Organization (PO), Working Memory capacity (WM), and Processing Speed (PS).

### 2.1. Factor-Analytical Approaches

**Figure 1:**
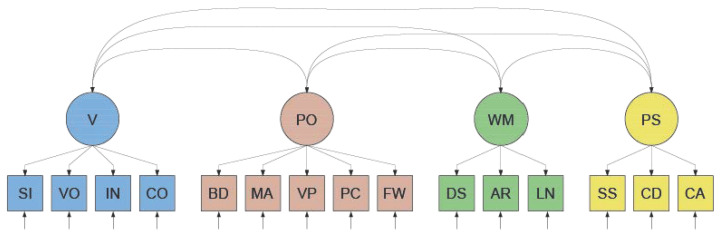
WAIS–IV Measurement Model of intelligence. Note. In the measurement model of the Wechsler Adult Intelligence Scale—Fourth Edition (WAIS–IV, Wechsler 2008) the four constructs V = Verbal Ability ; PO = Perceptual Organization; WM = Working Memory capacity; PS = Processing Speed are defined as (possibly correlated) common sources of variance in the observed variables. These observed variables are the scores on the subtests SI = Similarities; VO = Vocabulary; IN = Information; CO = Comprehension; BD = Block Design; MA = Matrix Reasoning; VP = Visual Puzzles; PC = Picture Completion; FW = Figure Weights; DS = Digit Span; AR = Arithmetic; LN = Letter Number Sequencing; SS = Symbol Search; CD = Coding; CA = Cancellation. Subtest unique sources of variance are also present (upward arrows).

In the factor analytic tradition, these constructs—V, PO, WM, and PS—are hypothesized to represent common sources of the variance in the observed subtest scores. This is shown in the so-called WAIS-IV measurement model in Figure 1. To evaluate a measurement model, one can conduct a confirmatory factor analysis. In the present example, this would involve regressing the 15 observed variables—the WAIS-IV subtest scores—on the four unobserved variables (“factors” or “latent variables”) representing the common sources V, PO, WM, and PS according to their hypothesized pattern (see Table 1). This factor analysis yields multiple types of information, including the model’s fit statistics and parameter estimates. The fit statistics provide information on the extent to which the variance–covariance structure of the observed variables *as implied by the model* matches the variance–covariance structure among the variables *as observed*. To evaluate this extent, a variety of fit criteria have been developed (for an overview, see, e.g., Schermelleh-Engel et al. 2003). If (and only if) the model fit is adequate according to these criteria, one can assign an interpretation to the parameter estimates. These estimates include the point (and interval) estimates of the regression coefficients of the observed variables on the latent variables (commonly referred to as “factor loadings”), the residual variances in the observed variables, and the covariances or correlations among the latent variables.

In the factor analysis of empirical intelligence test data, the factor loadings and the correlations among the factors are generally positive and significant. This is consistent with the well-established finding that the observed correlations among indicators of cognitive abilities are predominantly positive (Carroll 1993), a phenomenon referred to as the “positive manifold” of intelligence (Thurstone 1935).

**Figure 2:**
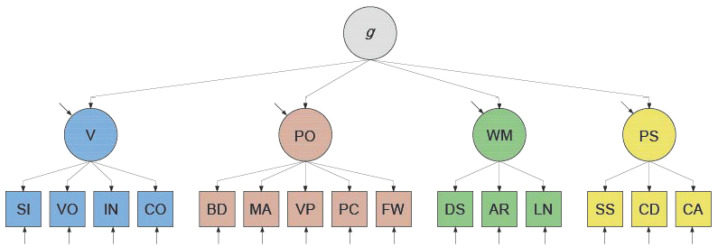
WAIS-IV Higher-order *g* Factor Model of Intelligence. Note. A higher-order *g* factor model explains the (positive) correlations among the factors in the measurement model by supposing a common dependency of those factors on the theoretical variable *g*. Variable *g* does not explain all of the variance in the factors, however, as indicated by the residuals (depicted as independent arrows that point to the factors). For abbreviations, see Figure 1.

To explain the positive correlations among the factors in the measurement model—and thus the positive manifold—Schmid and Leiman (1957) introduced higher-order factor modeling. This technique allows for an ultimate common dependence of the factors in the measurement model on one or more of the other factors (called higher-order factors), as visualized in Figure 2. That the correlations among the factors are imperfect is attributed to the presence of independent “residual” influences. Notably, higher-order factors have no observed indicators, unlike the factors that were already included in the measurement model and which are now referred to as “first-order” factors. Statistically, in our WAIS-IV example, the (first-order) factors V, PO, WM, and PS are regressed on a fifth (second-order) latent variable. Next, adhering to *g* theory (e.g., Jensen 1998), this second-order variable is interpreted as representing Spearman’s (1904) theoretical variable *g*, which stands for general intelligence. Although the exact nature of *g* remains unknown, it is hypothesized to be a single, unitary (non-cognitive, biological) variable that affects any test of cognitive performance (Jensen 1998). We refer to this theoretically grounded factor model as the (WAIS-IV) higher-order *g* factor model.

From the higher-order *g* factor model, it follows that the variance in the scores on each intelligence subtest can be decomposed into three orthogonal (i.e., statistically independent) variance components (Schmid and Leiman 1957): (1) a unique variance component, which includes, for example, variance due to pure measurement error, (2) a general component that is shared with all other subtests, due to their ultimate common dependence on *g*, and (3) a variance component that captures residual shared variance, meaning variance that is shared with some but not all of the other subtests. Such orthogonal variance decomposition was (originally) exactly what a bi-factor analysis aimed to accomplish (Holzinger and Swineford 1937).

**Figure 3:**
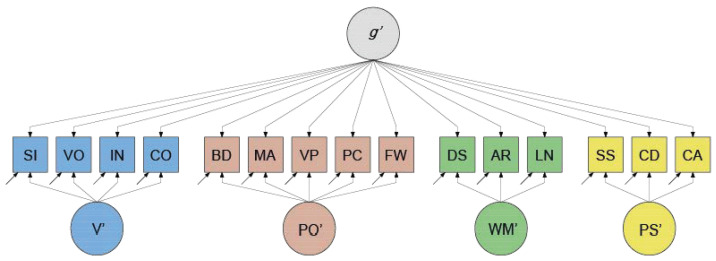
WAIS-IV Bi-factor decomposition of Intelligence. Note. A bi-factor model decomposes the variance in the scores on each subtest into three orthogonal variance components: (1) a unique variance component, which includes, for instance, variance due to pure measurement error; (2) a component that is shared with all other subtests (as visualized by a common dependence on variable *g*′); (3) a component that is shared with some but not all subtests (a visualized by dependencies on variables V′, PO′, WM′ or PS′). For abbreviations, see Figure 1.

Notably, a higher-order *g* factor model respecified as a bi-factor model includes a number of proportionality constraints on the factor loadings (Holzinger and Swineford 1937; Mansolf and Reise 2017; Schmid and Leiman 1957; Yung et al. 1999). These constraints result from the first-order factors in the higher-order *g* factor model mediating the effects of *g* on the subtest scores.^1^ The inclusion of proportionality constraints is not a requirement for the bi-factor model, however (Holzinger and Swineford 1937; Schmid and Leiman 1957; Yung et al. 1999). Relaxation of the constraints makes the bi-factor-model more flexible than the higher-order *g* factor model. A concrete example of an unconstrained bi-factor model of intelligence, pertaining to the WAIS-IV, is displayed in Figure 3. This shows that the scores on all subtests are directly regressed on a general latent variable (denoted *g*′), while the scores on the Verbal, Perceptual Organization, Working Memory, and Processing Speed subtests are additionally regressed on more narrowly defined latent variables (denoted here V′, PO′, WM′, and PS′). These more narrowly defined latent variables are all statistically independent of *g*′ and of each other, and are usually referred to as “group factors”.

As discussed extensively by, for instance, Hood (2008), Decker (2020), and Dolan and Borsboom (2023), the interpretation of the latent variables in a bi-factor decomposition is not as straightforward as in the measurement model and the higher-order *g* factor model. The interpretation also depends on the proportionalities of the factor loadings.

If the aforementioned proportionality constraints hold—that is, if the bi-factor model is merely a statistical respecification of the higher-order *g* factor model—then the following holds. The contribution of variable *g*′ to the variance in a given observed variable equals the contribution of the general factor to that observed variable *g* in the higher-order *g* factor model. Therefore, one could argue that variable *g*′ equals variable *g*. Indeed, variable *g* is a predictor of the performance on each subtest, albeit indirectly, as the higher-order *g* factor model shows. This is not detectable from the bi-factor decomposition model, which does not represent the causal model here, only the way the variance is decomposed. This distinction between the hypothesized causal model and the variance decomposition method is important with respect to variables V′, PO′, WM′, and PS′. These must have a different interpretation than the first-order factors V, PO, WM, and PS, which appear in the higher-order *g* factor model and measurement model. After all, the variables in the bi-factor model are independent of *g*′ (= *g*) and of each other, while the first-order factors in the higher-order factor model are not, due to their common dependence on *g*. Rather, the variables V′, PO′, WM′, and PS′ can be interpreted as the *residuals* of the factors V, PO, WM, and PS in the higher-order *g* factor model. These residuals are, indeed, also (indirect) predictors of the performance on certain subtests and are, indeed, independent of *g*. The factors V, PO, WM, and PS that figure in the measurement model and higher-order *g* factor model do not figure in the bi-factor decomposition model (but they should not be ignored, as their presence as mediators is the source of the grouping and of the proportional factor loadings).

In the absence of the proportionality constraints, when the factor loadings are all freely estimated, *g*′ is no longer identical to *g* in the higher-order *g* factor model, and the interpretative status of the variables V′, PO′, WM′, and PS′ becomes unclear (Hood 2008). When considering the bi-factor model as an alternative measurement model (rather than a method for decomposing the observed variance into variance components), it is important to note that a bi-factor model differs substantively from both the original measurement model and the higher-order factor model. For example, whereas in the measurement model and the higher-order factor model each subtest indicates a single latent variable (e.g., V), in a bi-factor model interpreted as a measurement model, the subtests are no longer unidimensional, but two-dimensional; each subtest now indicates two variables (i.e., *g*′ and e.g., V′). By definition, measurement invariance (Mellenbergh 1989) does not hold, as Hood (2008) points out: “[M]easurement invariance with respect to [*g*′] must be violated if one takes the test specific factor to be the group variable, and measurement invariance with respect to the test specific factor must be violated if one takes [*g*′] to be the group variable.”. That measurement invariance does not hold implies that, whatever the intelligence test measures (Van der Maas et al. 2014), “intelligence” will have a different meaning for different individuals or groups of individuals (Mellenbergh 1989). From the previous, it has been concluded that unconstrained bi-factor models of intelligence are inconsistent with *g* theory (Hood 2008), which implies that these models are theoretically weaker than higher-order *g* factor models; only the latter are consistent with *g* theory.

Typically, bi-factor models are of the unconstrained kind. As mentioned in the introduction, these tend to outperform higher-order *g* factor models. This holds in general and for the WAIS in particular (Cucina and Byle 2017). These results mean that the constraints implied by higher-order *g* factor models are untenable and, using Popperian logic, this is reason to reject this model. The next question becomes whether the rejection of higher-order *g* factor model is reason enough to adhere to a bi-factor model. This is doubtful: Apart from the conceptual difficulties with bi-factor models of intelligence (Decker 2020; Dolan and Borsboom 2023; Hood 2008) and the (potential) statistical bias against the higher-order *g* factor model (Morgan et al. 2015), recent studies into the correlational structure of the WAIS (Kan et al. 2019, 2020; Schmank et al. 2019) have demonstrated that bi-factor models fit worse than psychometric network models. What do such network models look like?

### 2.2. A Network Approach

Psychometric network modeling (Borsboom et al. 2021; Epskamp et al. 2018, 2017) can be viewed as an alternative to factor modeling in the sense that psychometric network models also aim to describe or explain the variance–covariance structure of individual differences. They do so without the need to invoke latent variables. The idea behind these models is that the variables included in the network model directly influence each other and, as a result, are correlated. With respect to the positive manifold, psychometric network models of intelligence are thus supported by the mutualism theory of intelligence (Van der Maas et al. 2017, 2006). This theory explains the positive manifold as the result of the dynamic interactions between cognitive abilities that take place during their development. Empirical evidence for such interactions exists, both within and between cognitive domains. An example within cognitive domains is that the growth of one mathematical skill improves the other mathematical skill (Hofman et al. 2018). An example between cognitive domains is that one’s increasing vocabulary benefits matrix reasoning (and vice versa) (Kievit et al. 2017). In the mutualism theory of intelligence, and more generally speaking in psychometric network models, not every variable needs to exert an influence on all other variables; interactions can be sparse, implying that effects can also be indirect. Furthermore, the interactions do not need to be strong and are not necessarily always bi-directional or symmetric. Some interactions may even be negative. As long as the interactions are predominantly positive, a positive manifold is expected (Van der Maas et al. 2006). When the interaction strengths differ across abilities, for example within and between domains, a clustered organization can emerge.

In the jargon of psychometrics, a psychometric network is a constellation of “nodes” and “edges” (Epskamp et al. 2017). Nodes are synonymous with observed variables—in our example, the WAIS intelligence subtest scores. Edges represent the relations among the nodes and are typically modeled as partial correlations (i.e., the correlation between two variables, after regressing for the effects of all the other variables included in the model). Non-significant edges are usually constrained to zero (Epskamp et al. 2017), so that network models, like factor models, have a certain number of degrees of freedom and can be put to the statistical test (e.g., Bulut et al. 2021; Kan et al. 2019, 2020; Schmank et al. 2019).

**Figure 4:**
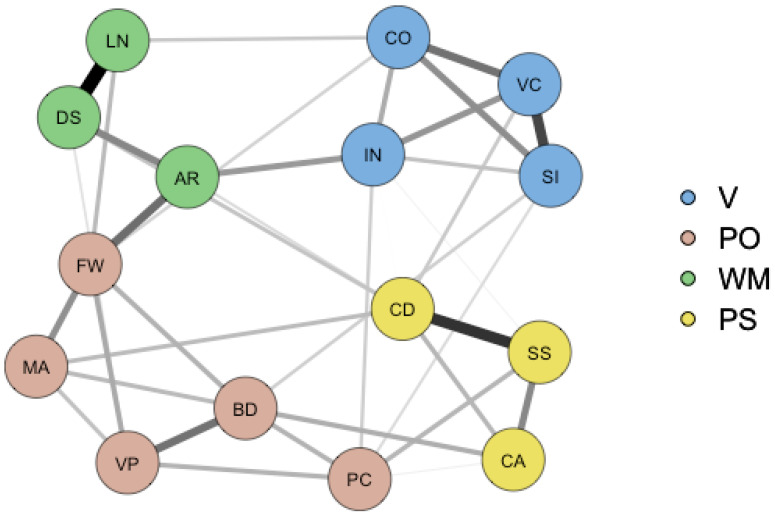
WAIS-IV psychometric network model of intelligence. Note. A graphical representation of the psychometric network model that was used in the present study. The nodes represent the WAIS-IV subtest scores, while the edges are partial correlations between the subtests. The stronger the partial correlation between two nodes, the thicker the edge that connects them. For abbreviations, see Figure 1.

Visualizations of the partial correlational structure of intelligence (e.g., Van der Maas et al. 2017, and Figure 4) typically show that the nodes cluster together. These clusters can be interpreted as the broader cognitive constructs, such as verbal ability, working memory, and so on. Adhering to the mutualism theory of intelligence, these clusters are emergent properties of the underlying dynamical system; they are abstractions and do not represent common sources of variance, as in the traditional factor analytic interpretation. Similarly, general intelligence is an emergent property (Van der Maas et al. 2014) and an abstraction, rather than an unobserved common source of variance, as the variable *g* is in *g* theory (Jensen 1998; Spearman 1904). The general factor that would be obtained in a factor analysis from data generated by a network or mutualistic mechanism would constitute a summary variable—and admittedly a sensible and useful one—but also one that has no instantiation in the real world. One may draw a parallel with a variable like “general health” (Kossakowski et al. 2016; Van der Maas et al. 2014). This variable also summarizes various correlated observations and is a result or outcome variable, rather than an underlying cause or source of variance in symptoms.

## 3. Present Study

The preceding discussion highlights that there are multiple ways to model the variance–covariance structure of intelligence. One way is to conduct a factor analysis, which may involve higher-order or bi-factor modeling. Another approach is network modeling. While all these models share similarities, they also differ, not least with respect to the hypothesized etiology of individual differences in the subtest scores. When fitted to a particular data set, the models may exhibit significant differences in terms of statistical fit. This underscores that fit statistics can be a valuable tool alongside theoretical considerations. Using Popperian logic, fit statistics enable researchers to reject certain models in favor of the remaining alternative models. Typically, model competitions involve two or more factor models (e.g., Major et al. 2012), but thanks to recent statistical advances, it is now possible to include psychometric network models in the model selection procedure (Kan et al. 2019, 2020; Schmank et al. 2019).^2^ This possibility, in turn, provides an opportunity to investigate the validity of the network hypothesis regarding why bi-factor models outperform higher-order *g* factor models. Moreover, we can conduct this investigation while controlling for the competing explanations that (1) bi-factor models represent the true data-generating mechanism, and (2) fit indices are biased against higher-order factor models in favor of bi-factor models. This is possible because these three explanations generate sets of predictions that are differential. We outline these sets of predictions below, after we detail how each explanation predicts the higher-order factor model’s outperformance using the bi-factor model.

**Explanation 1: The bi-factor model represents the true data-generating mechanism.** In general, the following holds: Provided that the true model is included in the set of competing models, and provided that fit measures behave as intended and do not produce biased results, this true model should yield better relative fit statistics than its untrue competitor(s). Models that include additional parameters are overly complex, and this extra complexity is penalized by relative fit statistics. Models that omit parameters are not complex enough and can be expected to show a worse relative fit. The empirical finding that bi-factor models outperform nested higher-order *g* factor models (Cucina and Byle 2017) is thus consistent, with the bi-factor model being the true model and the nested higher-order *g* factor model being an untrue competitor. The rejection of the higher-order *g* factor model is then justified because this model would concern an “oversimplification”.

**Explanation 2: Fit indices are inherently biased (in favor of bi-factor models and against higher-order *g* factor models).** If a higher-order *g* factor model is the true generating mechanism, a bi-factor model in which this higher-order *g* factor model is nested will contain a likelihood that is at least as good as that of the higher-order *g* factor model. In other words, whenever a higher-order *g* model fits the data in an absolute (exact or approximate) sense, a bi-factor model will also fit the data in an absolute (exact or approximate) sense. Relative fit criteria, however, penalize the addition of too many parameters. Thus, if these relative criteria work as intended and the penalty is adequate, the higher-order *g* factor model should emerge as the preferred model. If the penalty is not severe enough, the situation may arise where the model selection procedure suggests that the untrue bi-factor model should be preferred over the true higher-order *g* factor model. It then remains unclear to what extent this preference occurs. Notably, the possibility exists that fit indices are more generally biased, for example, in favor of the network model, and against the bi-factor and higher-order *g* factor model.

**Explanation 3: A non-nested network model underlies the empirical data.** In practice, the chances are high that the true model is not included in the set of competing candidate models. Thus, it is plausible that neither the fitted higher-order *g* factor model nor fitted the bi-factor model represents the true model. In this scenario, “unmodelled complexity” may be the source of the better relative fit of the bi-factor model (Kan et al. 2020; Murray and Johnson 2013): Since the likelihood of the higher-order *g* factor model cannot be higher than that of a bi-factor model in which it is nested, the bi-factor model can only capture more—never less—of such (additional, unmodelled) complexity (e.g., Morgan et al. 2015; Murray and Johnson 2013). In other words, whenever the true model is not included in the set of models, the bi-factor model is more likely to provide a good summary of the data than a higher-order *g* factor model. The implication is that if the true underlying model is a network model, it makes sense that the bi-factor model tends to provide a relatively better summary of the variance–covariance structure generated by the true network model. If this network is not nested in the bi-factor model, implying that the network cannot actually be respecified as a bi-factor model, the remaining questions are (1) whether the fitted bi-factor model shows acceptable fit in an absolute or approximate sense, (2) whether a nested higher-order model shows acceptable fit in an absolute or approximate sense, and (3) how the relative fit statistics of the bi-factor model compare to those of the nested higher-order *g* factor model.

**Differential Predictions**. Although all three explanations are capable of predicting that bi-factor models outperform higher-order *g* factor models on the basis of relative fit, they make additional predictions. When these predictions are considered collectively, and provided the power to distinguish between the models is sufficient, these sets of predictions differentiate between the competing explanations.

- If Explanation 1—the bi-factor model represents the true data-generating mechanism—is correct (and Explanations 2 and 3 are not), then:
  1. Fit statistics will show excellent exact fit and therefore (near) perfect approximate and incremental fit for the bi-factor model;
  2. A comparison between the bi-factor and higher-order *g* factor model will reject the latter for being too simplistic, while
  3. A comparison among three models—the bi-factor, higher-order *g* factor, and non-nested network model—will judge the latter to be less adequate than the true bi-factor model, so that
  4. *the true bi-factor model is expected to outperform both the nested higher-order g factor model and the non-nested network model*.
- If Explanation 2—fit indices are inherently biased in favor of bi-factor models and against higher-order factor models—is correct, then:
  1. Exact, approximate, and incremental fit statistics may or may not show good or excellent fit for the higher-order *g* factor model if that is the true model, and, thus, for the bi-factor model, while;
  2. *in a comparison between the true higher-order g factor model and the untrue bi-factor model, the relative fit indices are expected to show an increased preference for the untrue bi-factor model* (e.g., higher than the nominal significance level when performing a loglikelihood ratio test).
- If Explanation 3—a non-nested network model underlies the empirical data—is correct (and Explanations 1 and 2 are not), then:
  1. Fit statistics will show excellent exact, approximate, and incremental fit for this true network model;
  2. Fit statistics for the bi-factor model may show acceptable fit (and possibly for the higher-order *g* factor model as well), but (near) perfect fit is unlikely;
  3. A comparison between the untrue bi-factor model and the untrue higher-order *g* factor model would reject the latter in favor of the former, because the bi-factor has more fit propensity than the nested higher-order *g* factor model, whereas;
  4. A comparison among the three models—the bi-factor, higher-order *g* factor, and true, nonnested network model—should show a preference for the true (i.e., network) model, such that
  5. *the bi-factor model is expected to outperform the higher-order g factor model, but not the true network model*.

In summary, a comparison between the higher-order *g* factor model and bi-factor model comparison can (in)validate each explanation, while an additional comparison between three models—a bi-factor model, a nested higher-order *g* factor model, and a non-nested network model—can discriminate between Explanation 1 and Explanation 3 provided statistical bias is absent (hence provided Explanation 2 is not true). In the first case, the bi-factor model ends up as the model of preference and, in the latter case, the network model is the model of preference.

## 4. Method

With the above differential sets of predictions in mind, and drawing from the methodologies of Morgan et al. (2015) and Murray and Johnson (2013), we conducted a Monte Carlo simulation study, employing a fully crossed, three-by-three research design. That is, first a bi-factor model (without equality constraints), a (nested) higher-order *g* factor model, and a (non-nested) network model served as the data-generating mechanisms in the population. Second, we fitted all three models to all data sets and obtained the fit statistics. The relative fit statistics were used in model selection procedures. These selection procedures comprised (i) pairwise comparisons of the relative fit, including a comparison between a higher-order *g* factor model and a bi-factor model, and (ii) a comparison of the relative fit of all three models.

### 4.1. Data Generation

To arrive at empirically plausible parameter values for the factor models used in our simulations, we fitted the higher-order *g* factor and bi-factor models depicted in Figure 2 and Figure 3 to the German WAIS–IV (15 × 15) correlation matrix (Petermann 2012). For the network model, we paralleled the procedure described in Kan et al. (2020): First, we used the R psychonetrics function ggm() to compute a (15 × 15) full partial correlation matrix from the (15 × 15) US WAIS–IV correlation matrix. We then pruned this matrix at α = 0.01 (the default in psychonetrics) and searched for further improvements using the psychonetrics function stepup(), which automatically adds the parameter with the largest modification index until modification indices are no longer significant. The adjacency matrix of this pruned (and improved) partial correlation matrix was defined as the configural network model. Next, we fitted this configural model (confirmatory) to the German WAIS–IV correlation matrix, thereby freely estimating the parameter values.^3^

The factor and network model implied correlation matrices (Table A1 and Table A2) served as input for R library MASS function mvrnorm. This function generates multivariate normally distributed sample data according to a given population correlation or covariance matrix. The sample size was set as equal to the German sample size (1425) and the number of replications was set to 1000, resulting in a total of 3 × 1000 = 3000 sample data sets (with *N* = 1425 each). On these 3000 data sets, we fitted the higher-order *g* factor model (henceforth HF), the bi-factor model (henceforth BF), and the network model (henceforth NW) using Maximum Likelihood Estimation.

### 4.2. Model Fit Criteria

To facilitate a comparison of the results from our simulations with the results found in the literature, we obtained the same fit statistics as those reported by Cucina and Byle (2017). These included exact fit, approximate fit, incremental fit, and relative fit statistics (see below). To enhance clarity in our descriptions, we employed commonly used evaluation criteria (Schermelleh-Engel et al. 2003).

The *exact fit* of the models was assessed through the $\chi^{2}$ statistic, accompanied by its corresponding degrees of freedom (*df*) and associated *p*-values. We performed $\chi^{2}$ tests (α = .05) and determined the rejection rates of the models (which should be close to α in case the model concerned a true model). To evaluate the *approximate fit*, we obtained the root–mean–square of approximation (RMSEA) and applied the following rules of thumb: RMSEA ≤ .05 indicates “good fit”, RMSEA = .05–.08 “adequate fit”, RMSEA =  .08–.10 “mediocre fit”, and RMSEA > .10 “unacceptable fit”. To evaluate the *incremental fit* of the models, we obtained the normed fit index (NFI), the Tucker–Lewis index (TLI; a non-normed fit index), and the comparative fit index (CFI). For the NFI, values ≥ .95—and for the TLI and CFI, values ≥ .97—indicated “good” fit. Additionally, TLI and CFI values between .95 and .97 indicated “acceptable fit”. All other incremental fit values indicated “unacceptable fit”. To evaluate *relative fit*, used in the model selection procedure, we adhered to the following strategies. Only in instances where nested models were compared (i.e., the BF model with the HF model), we conducted a likelihood ratio test (i.e., the ∆$\chi^{2}$ test; α = .05). In all cases, we obtained the AIC and BIC and adhered to the rule that lower values of these fit criteria indicate a better relative fit (Schermelleh-Engel et al. 2003). In each simulation run, the model with the lowest value was declared the preferred model.

### 4.3. Analysis

When analyzing the results of our simulations, we first checked the performance of all fit indices. This was relevant for the evaluation of Explanation 2 (fit indices are biased) but also for the evaluation of Explanations 1 and 3, both of which lean on the premise that relative fit indices are unbiased. This performance check is described in full detail in Appendix B.

If the fit measures behaved as expected or intended, with no bias against the HF model in favor of the BF model, Explanation 2 was rejected as a valid explanation for the observed superiority of bi-factor models over higher-order *g* factor models. Concerning Explanation 1 (the BF model is the true model), if the BF model was the true data-generating mechanism and the fit indices performed as intended, the ∆$\chi^{2}$ test, AIC, and BIC were expected to favor the BF model as the preferred model. If this expectation was met, Explanation 1 was considered valid. Regarding Explanation 3 (a network model is the true model), again, assuming the fit indices performed as intended, the ∆$\chi^{2}$ test, AIC, and BIC were expected to exhibit a tendency to choose the BF model as the preferred model in the BF–HF model comparison. If this expectation was met, Explanation 3 was considered valid.

In addition to verifying the *validity* of the three competing explanations, our objective was to assess the *plausibility* of the explanations that were deemed valid. To achieve this, we compared the fit results of the simulations with the fit results reported in empirical studies. Fit values for the bi-factor and higher-order *g* models needed to closely align with those reported in Cucina and Byle (2017, see also Table 2). For instance, we expected RMSEA values to fall within the “adequate” to “mediocre” range, rather than being close to 0 (“perfect fit”) or exceeding 0.10 (“unacceptable fit”).

### 4.4. Software

All simulations and analyses were conducted in R (R Core Team 2022), using the RStudio (RStudio Team 2022) interface. We used R packages MASS (Venables and Ripley 2002) for data generation, tidyverse for data wrangling and visualization (Wickham et al. 2019), and qgraph (Epskamp et al. 2021) to extract and display network structures. Confirmatory factor and network analyses were performed in R package psychonetrics (Epskamp 2021) and cross-validated in OpenMx (Boker et al. 2011). Here, we limit ourselves to reporting the results from psychonetrics. Codes and output are available on the Open Science Framework (OSF): https://osf.io/xp869/ (see also https://github.com/KJKan/pame_I).

## 5. Results

### 5.1. Performance of Fit Indices

As outlined in Appendix B, when the true model was the HF model, all fit statistics behaved as anticipated. Key findings include the following. In a pairwise comparison, the ∆$\chi^{2}$ test exhibited a rejection rate of the HF model in favor of the BF model in 5.90% (CI_95_=[4.56%,7.56%]) of cases, in alignment with the nominal significance level of 5%. The BIC consistently selected the true model 100% of the time, demonstrating perfect performance. Although not flawlessly, the AIC also performed well, showing a preference for the BF model in 3.30% of instances when the true model was the HF model. Since this percentage did not exceed the percentage associated with the ∆$\chi^{2}$ test, we concluded that the performance of the AIC did not indicate bias in favor of the BF model over the HF model. In a broader context, when the true model was part of the set of models being compared, the relative fit indices AIC and BIC (and ∆$\chi^{2}$, where applicable) successfully identified the true model approximately 95% of the time.

In conclusion, we found no evidence that fit measures were biased in general or that they were biased against the HF model in favor of the BF model in particular. In the context of the present simulation study, Explanation 2 was dismissed as a valid explanation for the observation that BF models outperform HF models. The absence of bias simplified the assessment of the remaining explanations.

### 5.2. Checking the Validity and Plausibility of Remaining Explanations

When the true model was the BF model, the HF versus BF comparison always showed a preference for the BF model, regardless of whether the ∆$\chi^{2}$ difference test or the incremental fit indices AIC or BIC were used. This result validated Explanation 1—the true model is the bi-factor model. We assigned a low level of plausibility to this explanation, however, because if Explanation 1 were true, the $\chi^{2}$-test is expected to show non-significant results (exact fit is tenable), and therefore approximate and incremental fit indices should show a perfect or near perfect fit. Contrary to this expectation, the empirical results from Cucina and Byle (2017) demonstrate that in reality, exact fit is not tenable, and approximate and incremental fit indices show “adequate” to “good fit”, rather than perfect or near-perfect fit (see Table 2).

When the true model was the NW model, the HF versus BF model comparison consistently showed a preference for the BF model, regardless of whether the ∆$\chi^{2}$ difference test or the incremental fit indices AIC or BIC were used. This result validated Explanation 3. We considered the plausibility of this explanation to be high. As detailed in Appendix B, the exact, approximate, and incremental fit statistics obtained from the NW simulations were comparable to those reported in the literature: both our results and the results in Cucina and Byle (2017) show that (1) obtaining an exact fit for the factor models is untenable and (2) approximate and incremental fit indices display an imperfect fit. Moreover, the average fit values of the BF and HF models when the true model was the NW model were numerically closer than in the situation when the BF model was the true model.

### 5.3. Conclusions

Based on the results of our simulations, we deemed Explanation 2 (fit indices are biased) to be invalid. Both Explanation 1 (the BF model is the true model) and Explanation 3 (the NW model is the true model) were judged to be valid. The plausibility of Explanation 1 was considered low, while that of Explanation 3 was high, or at least higher than that of Explanation 1.

## 6. Discussion

Using Monte Carlo simulation, we examined the validity and plausibility of three competing explanations for why bi-factor models of intelligence outperform higher-order factor models of intelligence. These explanations were as follows: (1) the bi-factor model is the true model, (2) fit indices are biased against the higher-order *g* factor model, and (3) the true model is a network model. We found no evidence for the second explanation: all fit statistics behaved as expected and the relative fit statistics worked as intended. The absence of statistical bias simplifies the evaluation of the two remaining explanations.

The empirical observation that bi-factor models outperform higher-order *g* factor models in over 90% of cases (Cucina and Byle 2017) aligns with the hypothesis that a bi-factor model, rather than a higher-order *g* factor model, represents the true underlying structure. However, the plausibility of this hypothesis is low: when a true model is fitted to the data, exact fit measures should reject this model only about 5% of the time, while approximate and incremental fit measures should indicate a fit that is perfect or near-perfect. Empirical results, as shown in Table 2, reveal that this is not the case. The implication is that there must be some model misspecification in the reported bi-factor models, resulting in an imperfect fit. This misspecification may be local or global, but small, making an exact fit unattainable while approximate fit remains acceptable. Nonetheless, these findings suggest that there is additional complexity or inadequacy, which is not captured by the specified bi-factor models. This misspecification only highlights the possibility that the true data-generating mechanism is a network (Explanation 3).

This network explanation was validated by directly comparing the bi-factor model with the higher-order *g* factor model when the true model was a non-nested network model. In this scenario, the bi-factor model was consistently selected as the preferred model. We also deemed the network explanation plausible, as the results from our network simulations closely align with the fit values reported in the literature. At least, the plausibility of the network explanation is higher compared to the explanation that the bi-factor model represents the true underlying data-generating mechanism, as the latter would predict a perfect or near-perfect fit for the bi-factor model, which is in contrast with the empirical findings.

### 6.1. Limitations

In the end, the true data-generating mechanism can never be uncovered by statistical analysis alone, since one can always come up with equivalent models, that is, models with an identical or comparable fit (MacCallum et al. 1993). Given a table such as Table 2, proponents of a higher-order *g* factor model may argue that the possibility remains that a higher-order factor model represents the true underlying data-generating model, albeit a different one than was considered. For instance, the possibility remains that certain cross-loadings or correlated error terms were not specified. Likewise, given the imperfect fit that was observed, advocates of bi-factor models may argue that it is still possible that a bi-factor model, rather than a network model, underlies the data, but, again, a different or more complex bi-factor model than was considered. We call for more simulation studies, in line with the setup of Murray and Johnson (2013), in which the true model is not included in the set of candidate models. In this way, one can not only examine whether untrue bi-factor models tend to outperform untrue higher-order factor models, but also whether untrue factor models tend to be outperformed by untrue network models. In other words, such a setup would allow for the investigation of the fit propensities (Falk and Muthukrishna 2021) of network models.

Because our study was restricted to a particular intelligence test battery (i.e., the WAIS–IV), we also call for future simulations based on the psychometric properties of other test batteries that have undergone factor and network analysis, for instance, the Woodcock–Johnson (McGrew et al. 2023; Schrank and Wendling 2018). In such simulation studies, we recommend, for example, the exploration of parameter values. Although *the principle* behind network models is supported by theory (Savi et al. 2021; Van der Maas et al. 2017, 2006), whether (all) the specific parameter values are meaningful is a separate matter, which still requires critical evaluation. This issue extends beyond network models of intelligence and applies broadly, including network models in the domain of psychopathology (Borsboom 2022). While psychometric network modeling holds promise, it is also important to acknowledge that current network models of intelligence and psychopathology are in an early stage of development. We argue that this actually holds true for bi-factor models of intelligence (and psychopathology) as well, because bi-factor models are incongruent with *g* theory (Decker 2020; Hood 2008) (*p* theory within the field of psychopathology; Dolan and Borsboom 2023). Thus, to date, it remains uncertain whether the latent variables and parameters in bi-factor models of intelligence (and psychopathology) can be interpreted in a meaningful way.

### 6.2. Strengths

**Figure 5:**
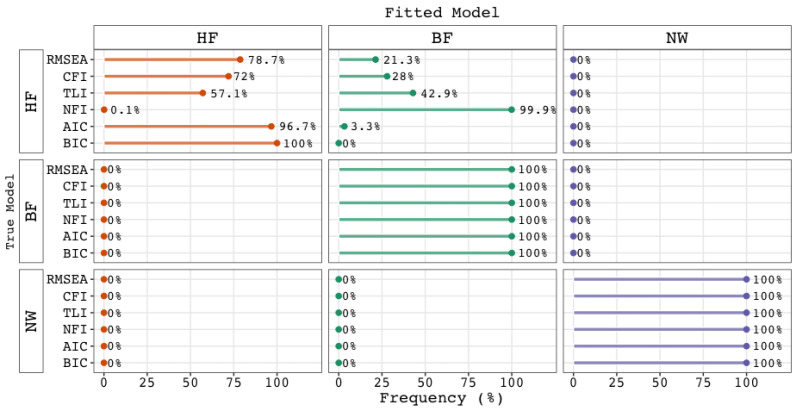
Preference for a Model in a Statistical Comparison Among Three Competing Models when Approximate (RMSEA), Incremental (NFI, TLI ,CFI), or Relative (BIC and AIC) Fit Statistics are Used. Note. The rows indicate which model generated the data, while the columns and colors represent the fitted model. Abbreviations: BIC = Bayesian Information Criterion; AIC = Akaike’s Information Criterion; NFI = normed fit index; TLI = Tucker-Lewis index; CFI = comparative fit index; RMSEA = root–mean–square error of approximation; BF = bi-factor model; HF = higher-order *g* factor model; NW = psychometric network model.

Substantive theory has formed (valid) reasons (e.g., Jensen 1998; Murray and Johnson 2013) to prefer higher-order *g* factor solutions over bi-factor solutions. Of course, the better fit of bi-factor models (Cucina and Byle 2017), should be taken into account, but it should not dominate the discussion and caution is necessary (e.g., Morgan et al. 2015). Researchers have alerted fellow researchers about the possibility that fit indices are biased against this model, favoring bi-factor models too easily. While we have found no evidence of bias in the fit indices themselves, we agree that biased inferences can arise from the comparison of fit statistics. We here note that exact, approximate, and even incremental fit indices serve as tools to help decide whether a model offers a satisfactory description or explanation of the observed variance-covariance structure. In our view, they are not intended to serve as *relative* fit measures, and therefore should not be used in model selection procedures. If one does so, however, the results can indeed yield biased inferences, as Figure 5 illustrates. From this figure, one can determine that if we had used the NFI as if it were a relative fit index, we would have inferred that the bi-factor model outperformed the higher-order *g* factor in more than 99% of the comparisons, when, in fact, the true model was a higher-order *g* factor model. Although not as extreme as the NFI, the use of the TLI, CFI, and RMSEA as relative indices would also have led to biased inferences, notably when nested models are compared. These results underscore our position that model selection procedures should rely on *relative* fit indices.

**Figure A6:**
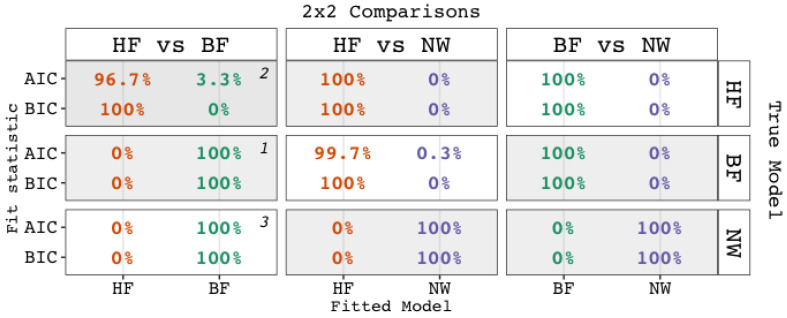
The Preference for a Model in a Statistical Comparison Between Two Competing Models when the Fit Statistics BIC and AIC were used. When the true model was a network, an HF vs. BF comparison favored the BF model, validating the network explanation; when the true model was a bifactor model, an HF vs. BF comparison favored the BF model, validating the bi-factor explanation; when a true model was included in a comparison, the true model was selected in more than 95% of the cases as being the preferred model, invalidating the statistical bias explanation. Note. Abbreviations: AIC = Akaike’s Information Criterion; BIC = Bayesian Information Criterion; HF = higher-order factor model; BF = bi-factor model; NW = network model. Shaded background: Comparisons in which the true model was included. White background: Comparisons in which the true model was not included. ^1^ Results relevant to Explanation 1—the bi-factor model is the true model. ^2^ Results relevant to Explanation 2—fit measures are biased (against the HF model in favor of the BF model). ^3^ Results relevant to Explanation 3—a network underlies the data.

We can imagine that researchers may harbor skepticism regarding the better relative fit of network models compared to factor models. Since network models often (but do not necessarily) contain a larger number of parameters than factor models, one might be inclined to assume that “because of the greater complexity” in terms of the number of estimated parameters network models will outperform factor models. That is, one might suspect that fit measures will be *biased* in favor of network models over factor models. However, as previous research (Kan et al. 2019) and the present simulation study (see Appendix B) have shown, there is no evidence of such bias. When the bi-factor model was the true model, a direct comparison between the bi-factor model and the network model showed that the AIC favored the network model in only 0.3% of cases (Figure A6). The BIC never favored the network model. Moreover, when the bi-factor model was the true model, a direct comparison between higher-order *g* factor model and the network model resulted in a preference for the more parsimonious, *too simplistic*, higher-order *g* factor model, rather than the ‘overly complex’ network model (in terms of number of parameters in the model). In other words, measures of relative fit, particularly the BIC, effectively guard against the inclusion of excessive complexity.

### 6.3. Conclusions

Our message concerning the use of fit statistics is two-fold. Firstly, if researchers believe that the true model is among those being compared, they should rely on relative fit indices (such as AIC or BIC), rather than approximate or incremental measures of fit (see also Murray and Johnson 2013). Secondly, if researchers entertain the idea that the true model is not within the set of models being compared—perhaps due to their adhering to the notion that “all models are wrong (but some are useful)”—they should be aware that more complex models generally exhibit a higher likelihood than nested, simpler models. In the latter scenario, the choice revolves around whether they prefer an untrue model that is relatively parsimonious or one that is relatively complex. This preference may be somewhat arbitrary, or based on theoretical considerations, or related to the researcher’s overarching objectives, such as predictive accuracy (see, e.g., Eid et al. 2018; Van der Maas et al. 2014).

Returning to the objectives of models of intelligence discussed in the introduction, we reiterate that, historically, higher-order factor models and bi-factor models have served distinct objectives. The objective of the higher-order factor modeling approach (Schmid and Leiman 1957) has been to explain the positive correlations between the factors in a first-order factor model, aligning models of intelligence with Spearman (1904)’s *g* theory. The objective of the bi-factor analysis was to decompose observed variance into variance components (Holzinger and Swineford 1937). As such, bi-factor analysis was used as a diagnostic tool to assess the quality of items or subtests within a test battery. These different objectives of higher-order factor modeling and bi-factor analysis are both beneficial to the field, as they both expand our knowledge about the structure of intelligence. However, we need to keep in mind that the latent variables obtained through bi-factor analysis are nothing beyond variance components, while—from a (*g*) theoretical perspective—the latent variables in a measurement or higher-order factor model represent hypothesized sources (‘causes’) of variance. These different types of latent variables should not be conflated (Van der Maas et al. 2014). Discerning the strong correlation between, for example, one’s verbal ability and general intelligence is directly detectable from a higher-order factor model, but not from a bi-factor model. This is especially true when the orthogonal group factor on which the verbal tests load shares the label “Verbal” with the first-order factor “Verbal” in the measurement and higher-order *g* factor models (see also, Van Bork et al. 2017). We concur with Decker (2020) that, if the objective is to *explain* the structure of intelligence, a bi-factor model should only be considered in a model selection procedure if the researcher genuinely believes it is a candidate (approximately) true model, supported by theory as much as its rivaling models. It is therefore worth noting that bi-factor models present more challenges in aligning with intelligence theories than higher-order *g* factor models and network models. Consequently, we advocate for the inclusion of network models in future model selection processes, if not instead then alongside bi-factor models. The superior fit of bi-factor models, compared to higher-order factor models, may stem from mutualistic processes underlying the data. A better fit of a psychometric network model would lend support for such scenario. The bi-factor modeling results would then summarize the variance–covariance structure without the bi-factor model providing an *explanation* of this structure (and the same would hold for the higher-order *g* factor model).

Although the mutualistic network approach to intelligence is now over a decade old, empirical tests to evaluate psychometric network models against factor models are relatively new. However, exemplary analyses exist (e.g., Bulut et al. 2021; Kan et al. 2019, 2020; Schmank et al. 2019). As Schmank et al. (2019) note, a fair evaluation would consist of comparing confirmatory, theoretical inspired models. We therefore call for more confirmatory network studies, both within and outside the field of intelligence.

As the present study has shown, network modeling not only can describe and explain the structure of individual differences in intelligence, but can offer new insights into longstanding debates. We believe that adding a network perspective can shed light on many robust yet puzzling findings that exist within the factor analytic literature, of which the outperformance of the higher-order *g* factor model by the bi-factor model is merely one example.

## Figures and Tables

**Table 1:** Description of the WAIS-IV Subtests.

Category	Subtest	Task Description
Verbal Ability (V)	Similarities (SI)	Explain the similarity between two words or ideas.
	Vocabulary (VO)	Identify pictures of objects or provide definitions of words.
	Information (IN)	Answer common knowledge questions.
	Comprehension (CO)	Respond to questions regarding social settings or popular notions.
Perceptual Organization (PO)	Block Design (BD)	Pattern-based puzzle solving based on a presented model (Timed).
	Matrix Reasoning (MA)	Choose the best-fitting puzzle for an arrangement of pictures.
	Visual Puzzles (VP)	Select three puzzle pieces that might complete the illustrated problem.
	Picture Completion (PC)	Choose the missing image component.
	Figure Weights (FW)	Solve equations with objects instead of numbers.
Working Memory (WM)	Digit Span (DS)	Listen to numerical sequences and repeat them in a certain order.
	Arithmetic (AR)	Solving mathematical word problems spoken orally (Timed).
	Letter–Number Sequencing (LN)	Recall a sequence of numbers or letters in a given order.
Processing Speed (PS)	Symbol Search (SS)	Determine if a symbol corresponds to any of the symbols in a given sequence.
	Coding (CD)	Utilize a key to transcribe a code of digits (Timed).
	Cancellation (CA)	Cancel out objects of a given collection according to the instructions (Timed).

Note: A short description of each subtest of the Wechsler Adult Intelligence Scale—Fourth Edition (WAIS-IV).

**Table 2:** Summary of Fit Statistics from Past Bi-factor Research on the Wechsler Adult Scale of Intelligence (WAIS; adapted from Cucina and Byle 2017).

Study	Battery	Higher-Order Factor Model							Comparison		Bi-Factor Model						
		CFI	TLI	NFI	RMSEA	AIC	$\chi^{2}$	*df*	$∆\chi^{2}$	∆*df*	CFI	TLI	NFI	RMSEA	AIC	$\chi^{2}$	*df*
Gignac and Watkins (2013)	WAIS–IV	0.945	0.933	0.918	0.068	314.75	246.75 ***	86	99.47 ***	11	0.975	0.965	0.951	0.049	237.28	147.28 ***	75
Gignac and Watkins (2013)	WAIS–IV	0.959	0.950	0.944	0.064	366.51	298.51 ***	86	101.30 ***	11	0.967	0.967	0.963	0.052	287.21	197.21 ***	75
Gignac and Watkins (2013)	WAIS–IV	0.943	0.930	0.920	0.075	347.28	279.28 ***	86	118.85 ***	11	0.975	0.965	0.954	0.053	250.43	16.43 ***	75
Gignac and Watkins (2013)	WAIS–IV	0.948	0.937	0.927	0.074	341.93	273.93 ***	86	78.98 ***	11	0.967	0.954	0.948	0.063	284.95	194.95 ***	75
Gignac (2005)	WAIS-R	0.970	0.959	0.967	0.068	443.97	391.97 ***	40	229.69 ***	7	0.989	0.982	0.986	0.046	228.28	162.28 ***	33
Gignac (2006)	WAIS–III	0.968	0.959	0.965	0.064	723.38	663.38 ***	61	215.13 ***	10	0.979	0.968	0.976	0.056	528.25	448.25 ***	51
Golay and Lecerf (2011)	WAIS–III	0.965	0.956	0.957	0.059	359.50	301.50 ***	62	178.50 ***	9	0.990	0.985	0.983	0.035	199.00	123.00 ***	53
Niileksela et al. (2013)	WAIS–IV	0.964	0.967	0.942	0.067	193.62	179.62 ***	71	10.76 ^†^	5	0.966	0.966	0.945	0.062	192.86	168.86 ***	66

Note. Abbreviations: CFI = comparative fit index; TLI = Tucker–Lewis index; NFI = fit index; RMSEA = root-meansquare error of approximation; AIC = Akaike information criterion; *df* = degrees of freedom; WISC-IV = Wechsler Intelligence Scale for Children—Fourth Edition; WAIS–IV = Wechsler Adult Intelligence Scale—Fourth Edition; WAIS-R = Wechsler Adult Intelligence Scale—Revised Edition; WAIS-III = Wechsler Adult Intelligence Scale—Third Edition. ^†^ *p* ≤ .10. *** *p* < .001.

**Figure A1:**
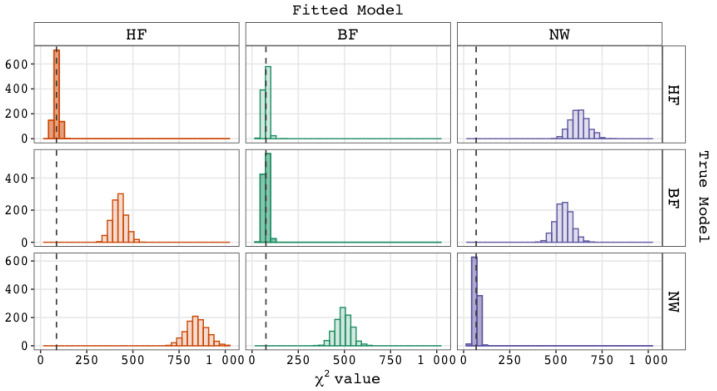
Distributions of the $\chi^{2}$ Statistic. Note. The plot shows the distribution of $\chi^{2}$-values within the nine conditions for the 1000 replications of the simulation design. The rows indicate which model generated the data, while the columns and colors indicate which model was fitted. The conditions in which a true model was fitted are on the diagonal and are shown in a darker shade. The dashed line represents the degrees of freedom of the fitted model (hence the expected $\chi^{2}$-values for the true model). Abbreviations: BF = bi-factor; HF = higher-order factor; NW = network.

**Figure A2:**
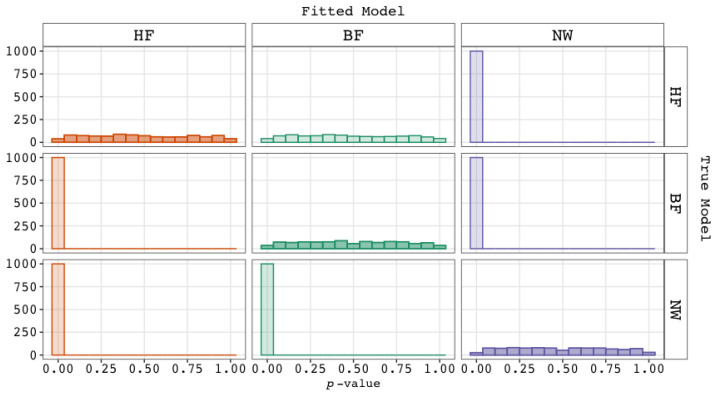
Distributions of the *p*-values Pertaining to the $\chi^{2}$ Statistic (see Figure A1). Note. The plot shows how the *p*-values of the $\chi^{2}$ statistic (see Figure A1) are distributed within the nine conditions. The rows indicate which model generated the data, while the columns and colors indicate which model was fitted. The conditions in which a true model was fitted are on the diagonal and are shown in a darker shade. Abbreviations: BF = bi-factor; HF = higher-order factor; NW = network.

**Figure A3:**
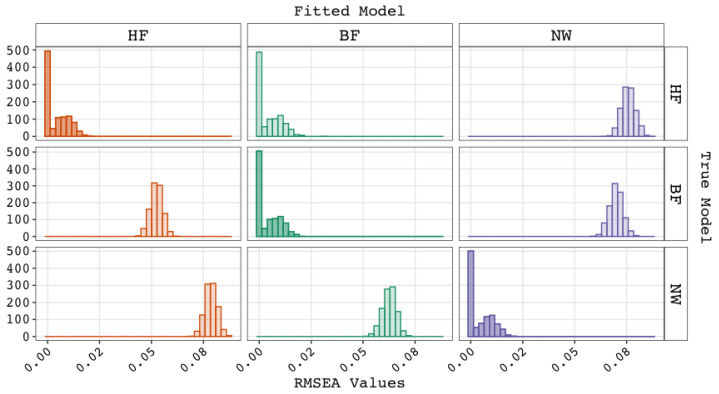
Distributions of Approximate Fit Values (RMSEA). Note. The graph shows the distribution of the RMSEA values within the nine simulation conditions for 1000 replications. The rows indicate which model generated the data, while the columns and colors indicate which model was fitted. The rows indicate which model generated the data, while the columns and colors indicate which model was fitted. The conditions in which a true model was fitted are on the diagonal and are shown in a darker shade. Abbreviations: BF = bi-factor; HF = higher-order factor; NW = network. Abbreviations: RMSEA = root–mean–square error of approximation; BF = bi-factor; HF = higher-order factor; NW = network.

**Figure A4:**
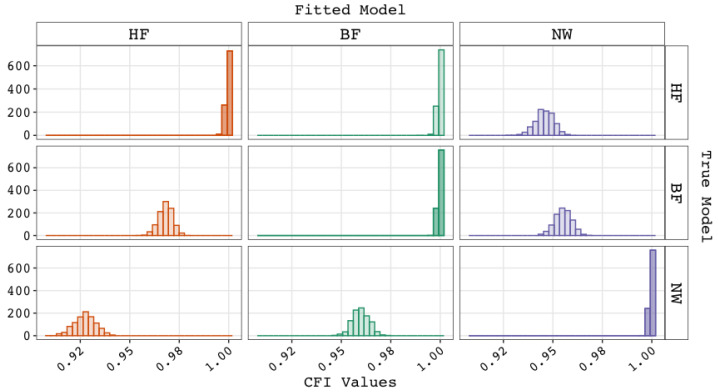
Distributions of Incremental Fit Values (CFI). Note. The graph shows the distribution of the CFI values for the 1000 replications within the nine simulation conditions. The rows indicate which model generated the data, while the columns and colors indicate which model was fitted. The conditions in which a true model was fitted are on the diagonal and are shown in a darker shade. Abbreviations: CFI = comparative fit index; BF = bi-factor; HF = higher-order factor; NW = network.

**Figure A5:**
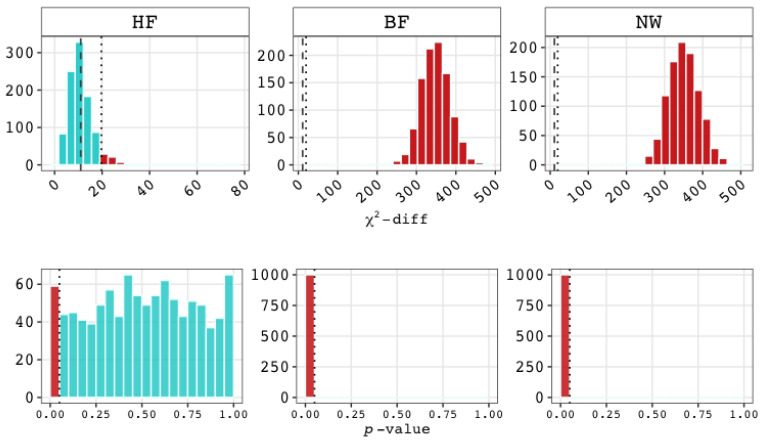
Distributions of the ∆$\chi^{2}$ values (**top**) and corresponding *p*-values (**bottom**) when testing the higher-order factor model against the bi-factor model in three situations: When the true model is (1) the higher-order factor model (**left**), (2) the bi-factor model (**middle**), and (3) the network model (**right**). Note. For all comparisons, ∆*df* = 11 (represented by the dashed line). Significant results in red. The dotted line represents either the critical value (19.675) of the ∆$\chi^{2}$ test (**top**) or the significance level (.05) (**bottom**). Abbreviations: HF = higher-order factor model; BF = bi-factor model; NW = network model.

## Data Availability

Codes and output are available on the Open Science Framework (OSF): https://osf.io/xp869/ and GitHub https://github.com/KJKan/pame_I.

## Notes

1 When the WAIS–IV higher-order *g* factor model is respecified as a bi-factor model, the standardized loading on *g*′ of, for instance, subtest SI ($\lambda_{SI,g^{\prime }}$) would be equal to the standardized factor loading of V on *g* ($\gamma_{V,g}$) multiplied by the standardized factor loading of SI on V ($\lambda_{SI,V}$) in the higher-order *g* factor model: $\lambda_{SI,g^{\prime }}=\gamma_{V,g}\cdot \lambda_{SI,V}$. The standardized bi-factor loading on variable V′ ($\lambda_{SI,V^{\prime }}$) would also be equal to a constant multiplied by the standardized factor loading of SI on V, namely $\lambda_{SI,V^{\prime }}=\sqrt{\left(1-\gamma_{V,g}^{2}\right)}\cdot \lambda_{SI,V}$. If we define the ratio (proportion) of the factor loadings on the *g*′ and V′ as $p_{g^{\prime },V^{\prime }}=\frac{\lambda_{SI,g^{\prime }}}{\lambda_{SI,V^{\prime }}}$, then it holds that this ratio is equal to the ratio of the factor loadings on the *g*′ and V′ for the subtests VO, IN, and CO. Thus, the proportionality constraints in the variance decomposition would include $\frac{\lambda_{SI,g^{\prime }}}{\lambda_{SI,V^{\prime }}}=\frac{\lambda_{VO,g^{\prime }}}{\lambda_{IN,V^{\prime }}}=\frac{\lambda_{IN,g^{\prime }}}{\lambda_{SI,V^{\prime }}}=\frac{\lambda_{CO,g^{\prime }}}{\lambda_{CO,V^{\prime }}}$. Similarly, $\frac{\lambda_{BD,g^{\prime }}}{\lambda_{BD,PO^{\prime }}}=\frac{\lambda_{MA,g^{\prime }}}{\lambda_{MA,PO^{\prime }}}=\frac{\lambda_{VP,g^{\prime }}}{\lambda_{VP,PO^{\prime }}}=\frac{\lambda_{PC,g^{\prime }}}{\lambda_{PC,PO^{\prime }}}=\frac{\lambda_{FW,g^{\prime }}}{\lambda_{FW,PO^{\prime }}}$, $\frac{\lambda_{DS,g^{\prime }}}{\lambda_{DS,WM^{\prime }}}=\frac{\lambda_{AR,g^{\prime }}}{\lambda_{AR,WM^{\prime }}}=\frac{\lambda_{LN,g^{\prime }}}{\lambda_{LN,WM^{\prime }}}$, and $\frac{\lambda_{SS,g^{\prime }}}{\lambda_{SS,PS^{\prime }}}=\frac{\lambda_{CD,g^{\prime }}}{\lambda_{CD,PS^{\prime }}}=\frac{\lambda_{CA,g^{\prime }}}{\lambda_{CA,PS^{\prime }}}.$ Instead of freely estimating 15+15=30 factor loadings, 15 loadings and 4 proportions are being estimated, giving an additional 11 degrees of freedom.

2 This possibility does not exist in most standard statistical software programs. As far as we know, a direct comparison is only possible in R (R Core Team 2022) using package OpenMx (Boker et al. 2011) or psychonetrics (Epskamp 2021).

3 We note that this network model lacks the rich history that the factor models have and that the use of the term “confirmatory” here is somewhat ambiguous; one might consider the method that was applied as an example of the *exploratory mode* of confirmatory techniques (Raykov and Marcoulides 2012). On the other hand, the confirmatory factor models of intelligence also originate from prior exploratory factor analyses conducted on other data sets and could also be viewed as cross-validations. Importantly, the different routes taken toward the parameter values do not affect the validity of the simulations or our argumentation. The essence of our simulation study is that, in order to evaluate the fit statistics of the network model effectively, the data generation should produce parameter estimates that are empirically plausible. This evaluates the fit statistics of the network model possible; hence, the provided fit statistics are not biased and, provided Explanation 3 is valid, the evaluation of the plausibility of this explanation is also unbiased. Furthermore, the fact that the configuration of the network model can be replicated across different samples strengthens the generalizability of our findings.

## Appendix A

**Table A1:** WAIS–IV intercorrelations as implied by the higher-order *g* factor model (lower triangle) and the bi-factor model (upper triangle).

	BD	SI	DS	MA	VO	AR	SS	VP	IN	CD	LN	FW	CO	CA	PC
BD	1.00	0.42	0.43	0.48	0.44	0.49	0.35	0.60	0.43	0.38	0.42	0.55	0.39	0.32	0.43
SI	0.44	1.00	0.42	0.43	0.71	0.48	0.35	0.38	0.60	0.38	0.42	0.48	0.66	0.31	0.35
DS	0.47	0.46	1.00	0.44	0.45	0.56	0.36	0.39	0.43	0.38	0.70	0.49	0.39	0.32	0.35
MA	0.50	0.41	0.44	1.00	0.45	0.50	0.36	0.46	0.44	0.39	0.43	0.52	0.40	0.32	0.38
VO	0.47	0.71	0.48	0.43	1.00	0.51	0.37	0.40	0.63	0.40	0.44	0.51	0.69	0.33	0.37
AR	0.43	0.42	0.59	0.40	0.44	1.00	0.41	0.44	0.49	0.44	0.51	0.56	0.45	0.37	0.40
SS	0.39	0.38	0.41	0.36	0.40	0.37	1.00	0.32	0.36	0.63	0.35	0.41	0.33	0.49	0.29
VP	0.51	0.42	0.44	0.47	0.44	0.41	0.37	1.00	0.39	0.35	0.38	0.53	0.35	0.29	0.43
IN	0.40	0.61	0.42	0.37	0.64	0.38	0.35	0.38	1.00	0.38	0.42	0.49	0.58	0.32	0.35
CD	0.40	0.39	0.41	0.37	0.41	0.38	0.62	0.38	0.35	1.00	0.38	0.44	0.35	0.46	0.31
LN	0.46	0.44	0.63	0.42	0.47	0.57	0.39	0.43	0.40	0.40	1.00	0.48	0.39	0.31	0.35
FW	0.57	0.46	0.49	0.53	0.49	0.45	0.41	0.53	0.42	0.42	0.48	1.00	0.45	0.36	0.44
CO	0.43	0.65	0.44	0.40	0.69	0.40	0.37	0.40	0.59	0.38	0.43	0.45	1.00	0.29	0.32
CA	0.31	0.30	0.32	0.29	0.32	0.29	0.48	0.29	0.27	0.49	0.31	0.32	0.29	1.00	0.26
PC	0.43	0.35	0.37	0.40	0.37	0.34	0.31	0.40	0.32	0.32	0.36	0.45	0.34	0.24	1.00

Note. BD = Block Design, SI = Similarities, DS = Digit Span, MA = Matrix Reasoning, VO = Vocabulary, AR = Arithmetic, SS = Symbol Search, VP = Visual Puzzles, IN = Information, CD = Coding, LN = Letter Number Sequencing, FW = Figure Weights, CO = Comprehension, CA = Cancellation, PC = Picture Completion.

**Table A2:** WAIS–IV intercorrelations as implied by the psychometric network model.

	BD	SI	DS	MA	VO	AR	SS	VP	IN	CD	LN	FW	CO	CA	PC
BD	1.00														
SI	0.42	1.00													
DS	0.35	0.34	1.00												
MA	0.48	0.30	0.33	1.00											
VO	0.38	0.72	0.36	0.30	1.00										
AR	0.40	0.39	0.56	0.37	0.41	1.00									
SS	0.34	0.30	0.31	0.30	0.32	0.31	1.00								
VP	0.59	0.34	0.37	0.46	0.32	0.39	0.29	1.00							
IN	0.37	0.59	0.37	0.30	0.63	0.50	0.31	0.33	1.00						
CD	0.38	0.37	0.43	0.40	0.42	0.42	0.63	0.34	0.37	1.00					
LN	0.34	0.34	0.70	0.31	0.36	0.47	0.26	0.34	0.35	0.36	1.00				
FW	0.55	0.41	0.48	0.53	0.41	0.60	0.32	0.54	0.43	0.41	0.49	1.00			
CO	0.38	0.66	0.39	0.31	0.69	0.43	0.29	0.34	0.60	0.37	0.42	0.46	1.00		
CA	0.39	0.25	0.25	0.27	0.26	0.26	0.49	0.28	0.25	0.46	0.22	0.29	0.24	1.00	
PC	0.46	0.38	0.28	0.30	0.35	0.31	0.38	0.43	0.38	0.35	0.26	0.36	0.34	0.30	1.00

Note. BD = Block Design, SI = Similarities, DS = Digit Span, MA = Matrix Reasoning, VO = Vocabulary, AR = Arithmetic, SS = Symbol Search, VP = Visual Puzzles, IN = Information, CD = Coding, LN = Letter Number Sequencing, FW = Figure Weights, CO = Comprehension, CA = Cancellation, PC = Picture Completion.

## Appendix B. Performance of Fit Indices

This appendix describes how we addressed (potential) bias in fit measures. We distinguished between (1) exact fit, as indicated by the $\chi^{2}$ test statistic, (2) approximate fit, as indicated by the Root Mean Square Error of Approximation (RMSEA), (3) incremental fit, as indicated by the Normed Fit Index (NFI), the Comparative Fit Index (CFI), and the Tucker–Lewis Index (TLI), and (4) relative fit, as indicated by the $\chi^{2}$ difference test statistic (only when nested models were compared) and Akaike’s Information Criterion (AIC) and the Bayesian Information Criterion (BIC).

### Appendix B.1. Expectations

We had the following expectations. Whenever we fit a true model, we expected the $\chi^{2}$ values to be distributed around its degrees of freedom (*df*), the associated *p*-values to have a uniform distribution, and, hence, using α=0.05, the rejection rate to be 5%. We then expected the approximate and incremental fit indices to indicate perfect or near-perfect fit (i.e., RMSEA values of 0 or close to 0, and NFI, TLI, and CFI values of 1 or close to 1).

Whenever we fit an untrue model, we expected the $\chi^{2}$ values to be distributed around a higher value than the number of degrees of freedom, the associated *p*-values to have a skewed distribution (to the right), and a rejection rate higher than 5%. The exception was when we fit the BF model while the true model was the nested HF model. Because of this nesting, we then expected, for both the BF and HF models, a uniform distribution of *p*-values, a rejection rate of 5%, and a perfect or near-perfect fit according to the RMSEA, NFI, TLI, and CFI.

When a true model was included in a model selection procedure, we expected the following. The AIC and BIC were expected to select the true model. If the true model was the bi-factor (BF) or the nested higher-order *g* factor (HF) model, and these models were tested against each other, the $\chi^{2}$ test was applicable. If the true model was the HF factor model, we expected these differences in $\chi^{2}$ values to be distributed around the difference in degrees of freedom between the models (∆df=11), and the associated *p*-values to have a uniform distribution. Furthermore, using a significance level of α=.05, we expected to reject the HF model (in favor of the BF model) about 5% of the time. If the BF factor model was the true model, we expected the *p*-values of the HF model to be skewed to the right, and thus a rejection rate of greater than 5% for the HF model.

When a true model was not included in a model selection procedure, we had no particular expectations, apart from the following: if the NW model were the true model, and the BF and nested HF were being compared, the AIC, BIC and $\chi^{2}$ test were expected to favor the BF model.

We defined the degree of bias in terms of the relative fit statistics favoring untrue competitor(s) over the true model. Thus, the degree of bias against the HF model in favor of the BF model was defined in terms of the relative frequency with which the ∆$\chi^{2}$ test and the information criteria AIC and BIC would select the BF model when the true model was the HF model. Bias was considered present if this frequency (the degree of bias) exceeded the nominal significance level of 5%.

If bias was present against the HF model in favor of the BF model, we considered Explanation 2 (the statistical bias explanation; see the main document) to be a valid explanation for the observation that bi-factor models outperform higher-order *g* factor models. If there was no bias against the HF model in favor of the BF model, then we considered Explanation 2 to be an invalid explanation—at least for present purposes.

### Appendix B.2. Results

Figure A1 shows the distributions of the $\chi^{2}$ statistics within each condition, and Figure A2 shows the distributions of the corresponding *p*-values. As expected, when a true model was fitted, the $\chi^{2}$-values were distributed around the number of degrees of freedom, while the associated *p*-values were uniformly distributed. In addition, when an untrue competitor was fitted, the $\chi^{2}$ values were generally much larger than the number of degrees of freedom, resulting in an abundance of significant results (*p*-values < .05). The exception was when we fitted the BF model and the true model was the HF model. As expected, the *p*-values were then also uniformly distributed.

Figure A3 shows the distribution of the approximate fit index RMSEA. As expected, and in general, we obtained (near) perfect fit values (values of 0 or close to 0) when fitting a true model. We also obtained (near) perfect fit values when fitting the BF model while the true model was the HF model, which was also as expected.

The distributions of the incremental fit indices CFI, NFI, and TLI showed similar patterns to the RMSEA (see Figure A4, which shows the CFI distributions). That is, the incremental fit values indicated (near) perfect fit when fitting a true model (and when fitting the BF model while the true model was the HF model).

Regarding the relative fit measures, we first address the comparison between the BF model and the HF model in the situation where one of them is the true model. As Figure A5 shows, when the BF model was the true model, the ∆$\chi^{2}$ test rejected the HF model and thus favored the true BF model in 100% of the time (implying that power was not an issue). When the true model was the HF model, the *p*-values were approximately uniformly distributed, and the ∆$\chi^{2}$ test resulted in a rejection rate of the HF model in favor of the BF model 5.90% of the time, which did not deviate from the nominal significance level of 5% ($\chi^{2}\;=\;1.521$, df=1, p=0.218, CI_95_=[4.56%,7.56%]). The BIC selected the true model 100% of the time, showing perfect performance (see Figure A6). The AIC also performed well, but not perfectly. It showed a preference for the BF model 3.30% of the time when the true model was the HF model. Since this percentage did not exceed the percentage associated with the ∆$\chi^{2}$ test, we judged this performance as not showing a true bias.

Comparing all three models, we found the following. Once again, the BIC showed perfect performance, selecting the true model 100% of the time. Again, the AIC also performed well, although it did not show perfect performance. When the true model was the HF model, it showed a preference for the BF model 3.30% of the time (but never for the NW model). In all other cases, the AIC selected the true model.

In summary, the exact, approximate, incremental, and relative fit measures performed as expected. We found no evidence that relative fit measures were biased in general, or that they were biased against the higher-order *g* factor model in favor of the bi-factor model in particular.
